# The clinical utility of circulating cell division control 42 in small-vessel coronary artery disease patients undergoing drug-coated balloon treatment

**DOI:** 10.1186/s12872-023-03476-5

**Published:** 2023-10-07

**Authors:** Lei Wu, Hui Li, Huanzhen Chen, Chunyu Fan, Yan Lu, Ruipeng Wei, Guangzhao Yang, Yongping Jia

**Affiliations:** Department of Cardiology, First Hospital of Shanxi Medical University, 85 Jiefang South Road, Taiyuan, 030001 Shanxi China

**Keywords:** Small-vessel coronary artery disease, Cell division control 42, Drug-coated balloon, Target lesion failure, Major adverse cardiovascular event

## Abstract

**Background:**

Cell division control 42 (CDC42) regulates atherosclerosis, blood lipids, and inflammation and thus affects coronary artery disease (CAD), but its utility in drug-coated balloon (DCB)-treated small-vessel CAD (SV-CAD) patients is unclear. This study intended to evaluate the change and prognostic role of CDC42 in SV-CAD patients underwent DCB.

**Methods:**

Serum CDC42 was measured by enzyme-linked immunosorbent assay in 211 SV-CAD patients underwent DCB at baseline, day (D) 1, D3, and D7, as well as in 50 healthy controls (HCs).

**Results:**

CDC42 was decreased in SV-CAD patients compared to HCs (*P* < 0.001), and it was negatively associated with total cholesterol (*P* = 0.015), low-density lipoprotein cholesterol (*P* = 0.003), C-reactive protein (*P* = 0.001), multivessel disease (*P* = 0.020), and American college of cardiology/American heart association type B2/C lesions (*P* = 0.039) in SV-CAD patients. Longitudinally, CDC42 decreased from baseline to D1 and then gradually increased to D7 (*P* < 0.001) in SV-CAD patients after DCB. Interestingly, high CDC42 (cut-off value = 500 pg/mL) at baseline (*P* = 0.047), D3 (*P* = 0.046), and D7 (*P* = 0.008) was associated with a lower accumulating target lesion failure (TLF) rate; high CDC42 at D3 (*P* = 0.037) and D7 (*P* = 0.041) was related to a lower accumulating major adverse cardiovascular event (MACE) rate in SV-CAD patients underwent DCB. Importantly, CDC42 at D7 (high vs. low) independently predicted lower accumulating TLF (hazard ratio (HR) = 0.145, *P* = 0.021) and MACE (HR = 0.295, *P* = 0.023) risks in SV-CAD patients underwent DCB.

**Conclusions:**

Circulating CDC42 level relates to milder disease conditions and independently estimates lower risks of TLF and MACE in SV-CAD patients underwent DCB, but further validation is still needed.

**Supplementary Information:**

The online version contains supplementary material available at 10.1186/s12872-023-03476-5.

## Introduction

Small-vessel coronary artery disease (SV-CAD) is characterized by the accumulation of atherosclerotic plaques in coronary vessels of relatively small caliber, which accounts for approximately 30% of symptomatic CAD patients [1, 2]. Currently, drug-eluting stent (DES) implantation and drug-coated balloon (DCB) have become important treatments for SV-CAD patients [3, 4]. However, DES may cause stent thrombosis and high risks of restenosis in CAD patients; particularly in SV-CAD patients with small vessel occlusion, it is even harder to operate [5, 6]. Compared to DES, DCB exhibits superior efficacy and safety in CAD patients, and it delivers antiproliferative drugs into the vascular wall without stent implantation, which makes it easier to operate in SV-CAD patients [6–8]. Nevertheless, some DCB-treated SV-CAD patients may still experience target lesion failure (TLF) and major adverse cardiovascular event (MACE) [9]. Therefore, it is vital to explore potential biomarkers to predict these events and improve the management of DCB-treated SV-CAD patients.

Cell division control 42 (CDC42) is a member of the Rho-guanosine triphosphatase family, which plays an important role in the progression of CAD by regulating atherosclerosis, inflammation, vascular recovery, endothelial barrier function, blood lipids, and so on [10, 11]. For example, one study suggests that CDC42 alleviates atherosclerosis by facilitating reendothelialization of injured arteries [12]. Another study shows that CDC42 inhibits inflammation by regulating T cell homeostasis [13]. Meanwhile, one previous study indicates that CDC42 induces endothelial regeneration and vascular recovery through the P21-activated kinase 1/protein kinase B pathway [14]. In the clinical field, recent studies suggest that CDC42 is decreased in CAD patients compared to controls, and serves as a biomarker to monitor the prognosis of CAD patients [10, 15]. The above studies reveal the potential of CDC42 as a biomarker for the management of CAD. However, the clinical value of CDC42 in DCB-treated SV-CAD patients remain unclear.

Therefore, this study intended to evaluate CDC42 variation during DCB treatment and its relationship with TLF and MACE rates in DCB-treated SV-CAD patients.

## Methods

### Subjects

A total of 211 SV-CAD patients who were treated with percutaneous transluminal coronary intervention (PCI) by DCB between October 2019 and October 2022 were consecutively included in this prospective, observational study. The inclusion criteria were as follows: (1) diagnosed with coronary artery disease by angiography; (2) had unstable or stable angina according to the guidelines [16, 17]; (3) elder than 18 years old; (4) had indications for PCI; (5) confirmed as SV-CAD, which was defined as a lesion diameter between 2.25 and 2.75 mm; (6) had only a single lesion in the target small vessel with stenosis of more than 70% (or more than 50% combined with symptoms of ischemia); and (7) planned to be treated with DCB. The exclusion criteria were as follows: (1) had an acute myocardial infarction (MI) or congestive heart failure; (2) a left ventricular ejection fraction of less than 35%; (3) underwent heart transplantation; and (4) pregnancy or lactation. In addition, a total of 50 healthy participants were enrolled as healthy controls (HCs), who were matched with the age and sex of SV-CAD patients. The research was approved by the Ethics Committee of First Hospital of Shanxi Medical University. Each subject signed the informed consent form.

### Collection of data and samples

Demographics, disease history, treatment history, disease characteristics, treatment information, and biochemical indexes were recorded and collected from the Electronic Medical System of our hospital. The collection was completed by one investigator and reviewed by another investigator. Coronary angiography was conducted by Digital Subtraction Angiography (Allura Xper FD20) from our hospital, and the imaging results were evaluated independently by two imaging specialists with more than 10 years of experience. If the evaluations did not agree, the two specialists discussed and unified the results. The hospital professionals conducted routine inspections of equipment Digital Subtraction Angiography every day in accordance with the operating guidelines; meanwhile, they cleaned and disinfected all parts of the equipment that had been touched. Meanwhile, the imaging equipment was calibrated regularly by the hospital’s dedicated personnel according to the standards of operating guidelines. All calibration procedures complied with regulations. Besides, peripheral blood samples were collected from SV-CAD patients at preoperation (baseline), 1st day (D1), 3rd day (D3), and 7th day (D7) of postoperation, as well as from HCs after enrollment. Then, serum in peripheral blood samples was isolated by centrifugation at 3500 revolutions per minute for 10 min and stored at -80℃ for the detection of CDC42. The CDC42 levels were measured by enzyme-linked immunosorbent assay (ELISA) conducted with ELISA kits (Jiangsu Jingmei Biotechnology Co., China) according to the manufacturer’s protocol. In brief, a total of 100 µl supernatant was added onto the CDC42 antibody-coated plate, and incubated at 25 °C for 2 h. After adding the biotin-conjugated detecting CDC42 antibody and incubating at 25 °C for 2 h, streptavidin-HRP was added and 3,3′-5,5′tetramethylbenzidine was used for development, which was incubated for 20 min at room temperature and protected from light. Then, CDC42 levels were measured by a multimode microplate reader (Synergy H1, BioTek, USA) at a wavelength of 450 nm. The professional personnel of the hospital equipment department carried out regular calibration of the enzyme label instrument according to the standards of operating guidelines, and all calibration procedures complied with regulations.

### Coronary angiography protocols

Coronary angiography was performed by Digital Subtraction Angiography (Allura Xper FD20) from our hospital. The operation process was as follows: The catheter was delivered to the coronary artery through the radial artery. Then, nonionic contrast agent (Ultravist) was injected by hand. The left coronary artery was injected with 8–10 ml of contrast agent, and the right coronary artery was injected with 6–8 ml of contrast agent. All injections were completed within two seconds. Exposure imaging was performed until coronary vein reflux. After obtaining the imaging, the catheter was removed from the radial artery, and the punctured artery was pressurized to prevent bleeding. After the procedure, the examined patients were taken to the recovery room for observation for 30–60 min and laid on their backs for a few hours. Pulse and blood pressure were carefully monitored for all patients during coronary angiography.

### Quality control measures

The quality control measures of our study were as follows: (1) Laboratory quality control measures: Each laboratory conducted testing in accordance with standard operating procedures and quality control procedures. (2) Qualification review of researchers: The professionals had clinical professional expertise, qualifications, and abilities, which were determined after the qualification review. All professionals were responsible for the whole project. (3) Training before clinical observation: All professionals had received rigorous training before the study to fully understand the specific connotation of the study’s protocol and all indicators. (4) Monitoring of patient adherence to medication observation: The drug counting method combined with the inquiry method was used to monitor patients’ adherence to medication, explanations were provided to patients, follow-up was strengthened, and good patient compliance was ensured. (5) Notification of possible adverse reactions during patient medication: Symptomatic treatment was performed in case of adverse reactions.

### Assessment

The SV-CAD patients received standardized follow-up until March 2023. The appearances of TLF and MACE were recorded. Target lesion failure comprised cardiovascular death, target lesion MI, or ischemia-driven target lesion revascularization [18]. MACE comprised cardiovascular death, MI in any lesion, or coronary revascularization in any lesion [18].

### Statistics

Based on the clinical experiment, we assumed that the MACE rates of SV-CAD patients with low and high CDC42 level was separately 0.30 and 0.10. Alpha was set at 0.05, and power was set at 0.85. The minimum sample size was calculated as 92. Considering a 15% dropout rate, the minimum sample size was 108. Then, we continuously enrolled as many patients as possible and eventually enrolled 211 patients. SPSS v26.0 (IBM, USA) and GraphPad Prism v8.01 (GraphPad Software Inc., USA) were adopted for data processing. Comparisons between groups were determined using the Wilcoxon rank sum test. Receiver operating characteristic (ROC) curves were generated to show the ability of CDC42 in distinguishing SV-CAD patients and HCs, as well as the ability of CDC42 in distinguishing patients with MACE from patients with non-MACE. The correlation of CDC42 with continuous variables was evaluated using Spearman correlation. The relationship between CDC42 and the categorical variables was analyzed using Kruskal-Wallis H rank sum test or Wilcoxon rank sum test. The correlation of CDC42 with the accumulating TLF rate and accumulating MACE rate was evaluated using Kaplan-Meier curves with the log-rank test, in which CDC42 levels were divided into low level (≤ 500 pg/mL) and high level (> 500 pg/mL) due to the baseline median CDC42 level of approximately 500 pg/mL. Factors related to TLF and MACE were screened using Cox’s regression models. The multivariate Cox’s regression model was established with step-forward mode, and all factors shown in the univariate model were included. *P* < 0.05 was considered to indicate significance.

## Results

### Baseline features of DCB-treated SV-CAD patients

The detailed baseline features of DCB-treated SV-CAD patients were exhibited in Table 1. The 211 enrolled DCB-treated SV-CAD patients had a mean age of 64.2 ± 10.1 years, including 53 (25.1%) females and 158 (74.9%) males. Meanwhile, there were 93 (44.1%) patients with previous PCI and 7 (3.3%) patients with previous coronary artery bypass grafting. A total of 103 (48.8%) patients had multivessel disease. Regarding the target vessel, there were 61 (28.9%) patients with left anterior descending artery (LAD) lesions, 111 (52.6%) patients with left circumflex artery lesions, and 39 (18.5%) patients with right coronary artery (RCA) lesions. Notably, 115 (54.5%) patients had American college of cardiology (ACC)/American heart association (AHA) type B2/C lesions. In terms of DCB treatment information, the mean diameter of DCB was 2.4 ± 0.1 mm, and the mean total length of DCB was 21.3 ± 5.7 mm. Additionally, the median [interquartile range (IQR)] values of low-density lipoprotein cholesterol (LDL-C), high-density lipoprotein cholesterol (HDL-C), and C-reactive protein (CRP) were 3.2 (2.6–3.9) mmol/L, 1.0 (0.8–1.2) mmol/L, and 5.5 (3.5–7.5) mg/L, respectively. More detailed clinical information on DCB-treated SV-CAD patients was exhibited in Table 1.

**Table 1:** Clinical characteristics of SV-CAD patients

Characteristics	SV-CAD patients (*N* = 211)
Age (years), mean ± SD	64.2 ± 10.1
Gender, No. (%)	
Female	53 (25.1)
Male	158 (74.9)
BMI (kg/m^2^), mean ± SD	25.4 ± 3.2
History of smoke, No. (%)	109 (51.7)
Hypertension, No. (%)	150 (71.1)
Hyperlipidemia, No. (%)	107 (50.7)
Diabetes mellitus, No. (%)	71 (33.6)
Family history of CAD, No. (%)	64 (30.3)
Previous MI, No. (%)	59 (28.0)
Previous PCI, No. (%)	93 (44.1)
Previous CABG, No. (%)	7 (3.3)
Clinical manifestation, No. (%)	
Stable angina	68 (32.2)
Unstable angina	143 (67.8)
Multivessel disease, No. (%)	103 (48.8)
Target vessel, No. (%)	
LAD	61 (28.9)
LCX	111 (52.6)
RCA	39 (18.5)
ACC/AHA type B2/C lesions, No. (%)	115 (54.5)
DCB diameter (mm), mean ± SD	2.4 ± 0.1
Total length of DCB (mm), mean ± SD	21.3 ± 5.7
WBC (10^^^9/L), median (IQR)	10.1 (7.8–12.6)
FBG (mmol/L), median (IQR)	5.5 (4.5–6.4)
Scr (µmol/L), median (IQR)	79.1 (69.7–90.3)
TG (mmol/L), median (IQR)	1.8 (1.0-2.4)
TC (mmol/L), median (IQR)	4.6 (3.9–5.3)
LDL-C (mmol/L), median (IQR)	3.2 (2.6–3.9)
HDL-C (mmol/L), median (IQR)	1.0 (0.8–1.2)
CRP (mg/L), median (IQR)	5.5 (3.5–7.5)

*SV-CAD* Small-vessel coronary artery disease, *SD *Standard deviation, *BMI *Body mass index, *CAD *Coronary artery disease, *MI *Myocardial infarction, *PCI* Percutaneous transluminal coronary intervention, *CABG *Coronary artery bypass grafting, *LAD *Left anterior descending artery, *LCX *Left circumflex artery, *RCA *Right coronary artery, *ACC* American college of cardiology, *AHA *American heart association, *DCB *Drug-coated balloon, *WBC *White blood cell, *IQR *Interquartile range, *FBG *Fasting plasma glucose, *Scr* Serum creatinine, *TG* Triglyceride, *TC *total cholesterol, *LDL-C *Low-density lipoprotein cholesterol, *HDL-C* High-density lipoprotein cholesterol, *CRP *C-reactive protein

### Comparison of CDC42 between DCB-treated SV-CAD patients and HCs

**Fig. 1:**
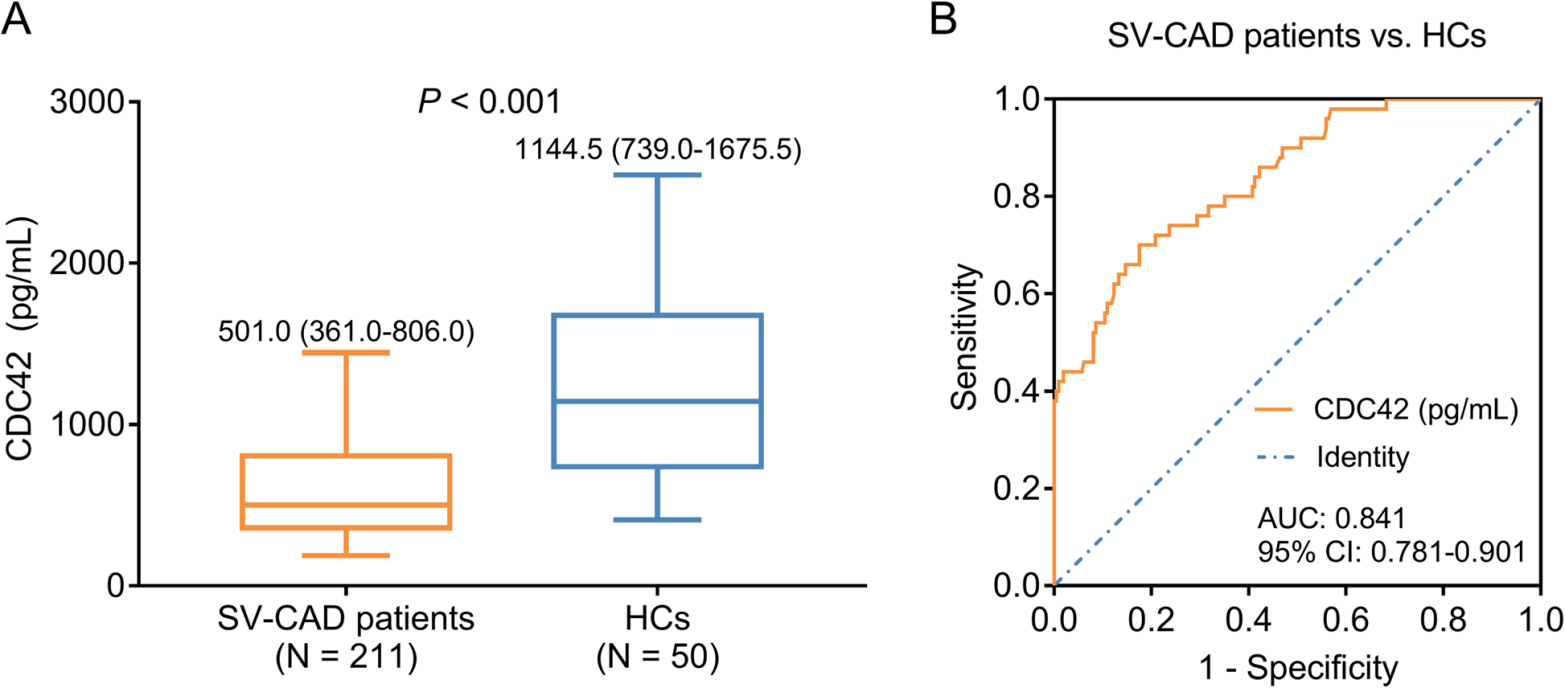
CDC42 in DCB-treated SV-CAD patients and HCs. Comparison of CDC42 between DCB-treated SV-CAD patients and HCs (**A**); ROC curve disclosing the value of CDC42 to differentiate DCB-treated SV-CAD patients from HCs (**B**)

CDC42 was reduced in DCB-treated SV-CAD patients compared to HCs [median (IQR): 501.0 (361.0-806.0) vs. 1144.5 (739.0-1675.5) pg/mL] (*P* < 0.001) (Fig. 1A). Moreover, CDC42 exhibited a good value in discriminating DCB-treated SV-CAD patients from HCs with the area under curve (AUC) of 0.841 [95% confidence interval (CI): 0.781–0.901] (Fig. 1B). In addition, the diagnostic characteristics of CDC42 between SV-CAD patients and HCs were shown in Supplementary Table 1. The values of sensitivity, specificity, accuracy, prevalence, Youden’s index, positive predictive value, and negative predictive value were 0.824645, 0.7, 0.800766, 0.808429, 0.524645, 0.920635, and 0.486111, respectively (Supplementary Table 1).

### Relationship of CDC42 with clinical features in DCB-treated SV-CAD patients

The correlation analyses of CDC42 with continuous characteristics and categorical characteristics in DCB-treated SV-CAD patients were shown in Tables 2 and 3, respectively. CDC42 was negatively associated with total cholesterol (TC) (*r*=-0.168, *P* = 0.015), LDL-C (*r*=-0.200, *P* = 0.003), and CRP (*r*=-0.236, *P* = 0.001) (Table 2). Furthermore, CDC42 was inversely related to multivessel disease (*P* = 0.020) and ACC/AHA type B2/C lesions (*P* = 0.039) (Table 3). However, CDC42 was not linked with other characteristics of DCB-treated SV-CAD patients, such as age, gender, clinical manifestation, etc. (all *P* > 0.05).

**Table 2:** Correlation of CDC42 with continuous characteristics in SV-CAD patients

Characteristics	*r*	*P* value
Age	-0.034	0.622
BMI	-0.099	0.152
DCB diameter	-0.010	0.883
Total length of DCB	-0.125	0.069
WBC	-0.127	0.066
FBG	-0.066	0.342
Scr	-0.093	0.177
TG	-0.094	0.176
TC	-0.168	0.015
LDL-C	-0.200	0.003
HDL-C	0.072	0.297
CRP	-0.236	0.001

*CDC42* Cell division cycle 42, *SV-CAD* Small-vessel coronary artery disease, *BMI *body mass index, *DCB *drug-coated balloon, *WBC *white blood cell, *FBG *Fasting plasma glucose, *Scr *Serum creatinine, *TG *Triglyceride, *TC *Total cholesterol, *LDL-C *Low-density lipoprotein cholesterol, *HDL-C* High-density lipoprotein cholesterol, *CRP* C-reactive protein

**Table 3:** Correlation of CDC42 with categorical characteristics in SV-CAD patients

Characteristics	CDC42 (pg/mL), median (IQR)	*P* value
Gender		0.211
Female	460.0 (360.0-679.0)	
Male	537.5 (360.0-819.8)	
History of smoke		0.218
No	577.0 (382.8–827.0)	
Yes	491.0 (354.5–734.0)	
Hypertension		0.124
No	592.0 (370.5-864.5)	
Yes	490.0 (356.8–764.0)	
Hyperlipidemia		0.068
No	573.5 (382.3–832.0)	
Yes	477.0 (354.0-754.0)	
Diabetes mellitus		0.439
No	530.0 (351.3-832.3)	
Yes	501.0 (383.0-706.0)	
Family history of CAD		0.397
No	491.0 (354.0-807.0)	
Yes	564.0 (388.3-805.3)	
Previous MI		0.150
No	519.0 (382.3-821.5)	
Yes	477.0 (329.0-706.0)	
Previous PCI		0.674
No	496.0 (364.0-828.8)	
Yes	501.0 (356.0-725.0)	
Previous CABG		0.617
No	505.5 (358.0-805.3)	
Yes	401.0 (383.0-971.0)	
Clinical manifestation		0.155
Stable angina	598.5 (368.0-862.8)	
Unstable angina	491.0 (356.0-795.0)	
Multivessel disease		0.020
No	572.0 (384.0-927.5)	
Yes	484.0 (345.0-731.0)	
Target vessel		0.880
LAD	561.0 (349.5–816.0)	
LCX	492.0 (380.0-819.0)	
RCA	500.0 (377.0-800.0)	
ACC/AHA type B2/C lesions		0.039
No	586.5 (386.3-901.3)	
Yes	477.0 (345.0-762.0)	

*CDC42* Cell division cycle 42, *SV-CAD *Small-vessel coronary artery disease, *IQR *Interquartile range, *CAD *Coronary artery disease, *MI *Myocardial infarction, *PCI *Percutaneous transluminal coronary intervention, *CABG *Coronary artery bypass grafting, *LAD *Left anterior descending artery, *LCX *Left circumflex artery, *RCA *Right coronary artery, *ACC *American college of cardiology, *AHA *American heart association

### CDC42 variation and its relationship with accumulating TLF and MACE rates in DCB-treated SV-CAD patients

**Fig. 2:**
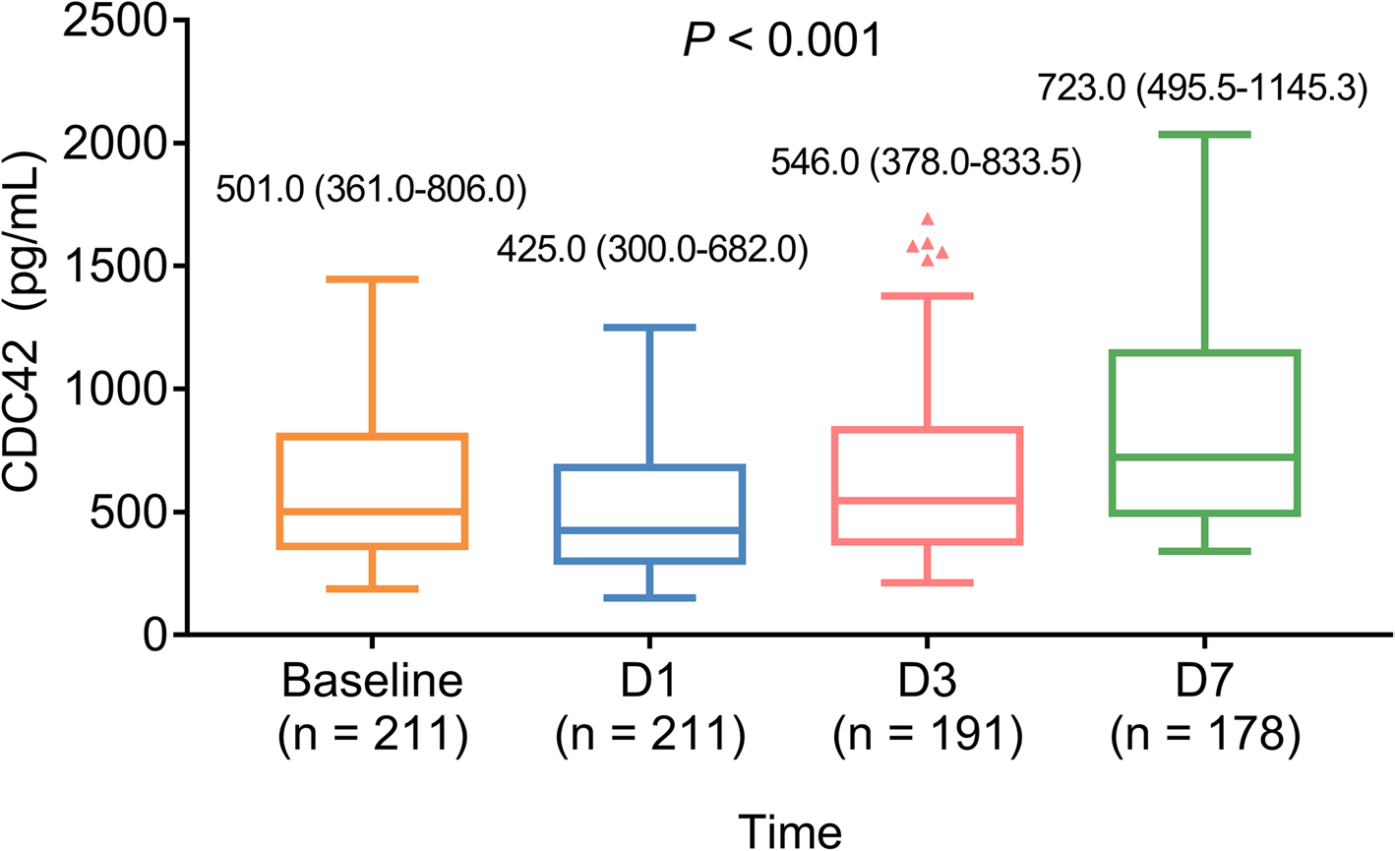
CDC42 variation from baseline to D7 in DCB-treated SV-CAD patients. The longitudinal change in CDC42 from baseline to D1, D3, and D7 in DCB-treated SV-CAD patients

CDC42 decreased from baseline to D1 and subsequently gradually increased from D1 to D7 in DCB-treated SV-CAD patients (*P* < 0.001) (Fig. 2).

From 2019 to 2022, the TLF rate was 10 (4.8%), and the incidence density of TLF was 4.6/100 person-year (PY). The MACE rate was 20 (9.5%), and the incidence density of MACE was 10.5/100 PY.

**Fig. 3:**
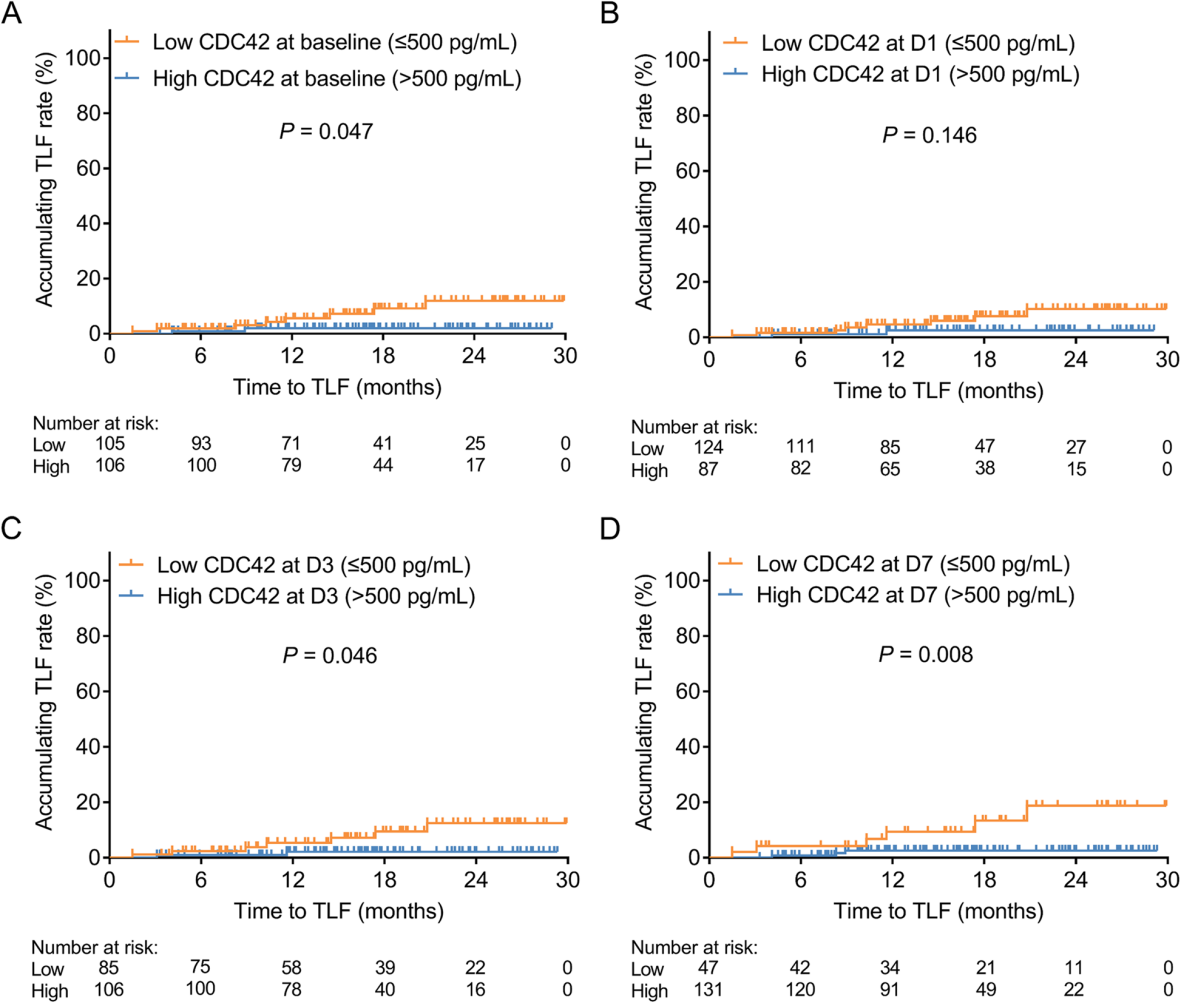
Correlation of CDC42 with the accumulating TLF rate in DCB-treated SV-CAD patients. The linkage of CDC42 at baseline (**A**), D1 (**B**), D3 (**C**), and D7 (**D**) with accumulating TLF rate in DCB-treated SV-CAD patients

Interestingly, high CDC42 at baseline was associated with a lower accumulating TLF rate in DCB-treated SV-CAD patients (*P* = 0.047) (Fig. 3A). Nevertheless, there was no linkage of CDC42 at D1 with the accumulating TLF rate in DCB-treated SV-CAD patients (*P* = 0.146) (Fig. 3B). Furthermore, high CDC42 at D3 (*P* = 0.046) and D7 (*P* = 0.008) was correlated with lower accumulating TLF rates in DCB-treated SV-CAD patients (Fig. 3C-D).

**Fig. 4:**
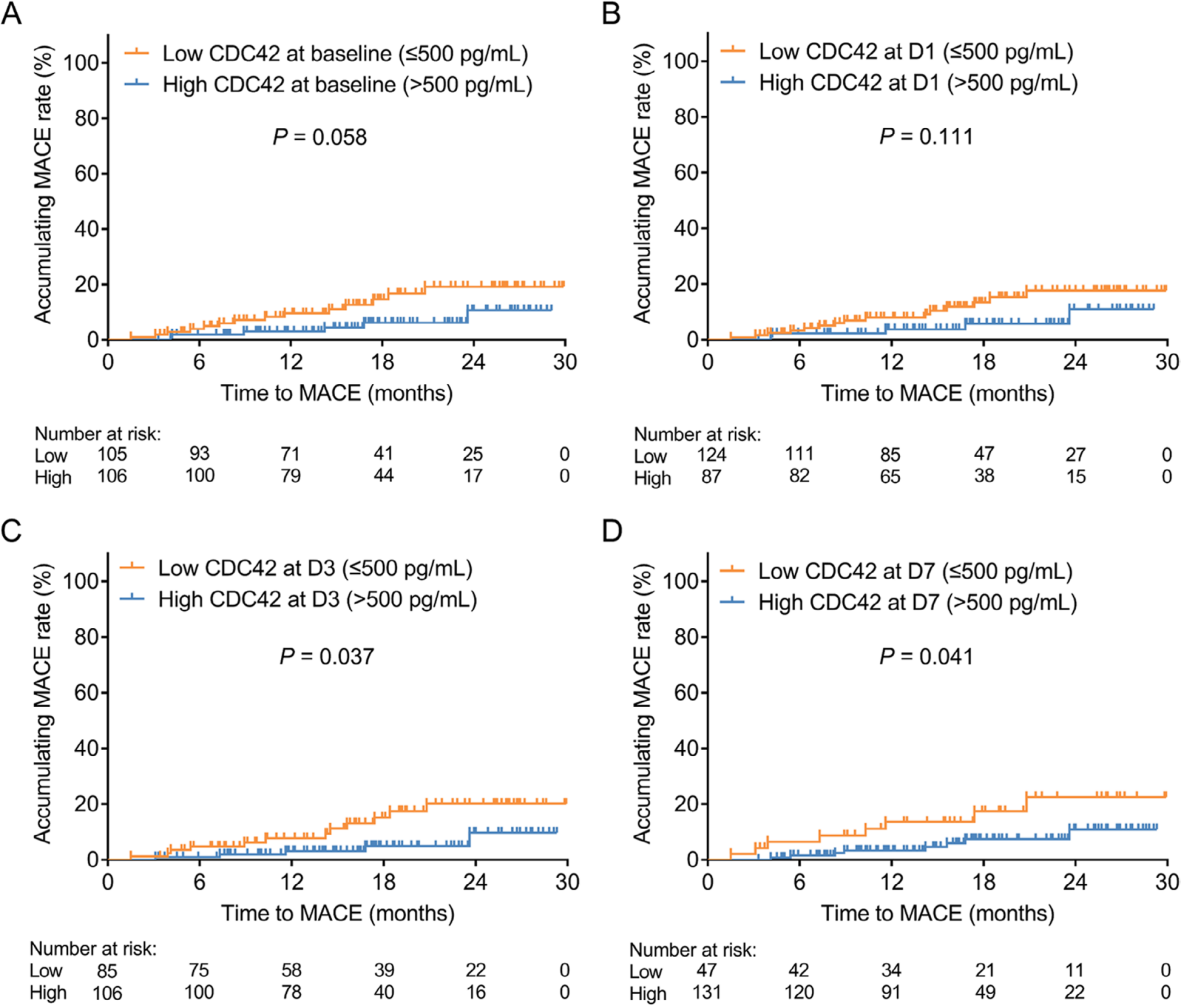
Correlation of CDC42 with the accumulating MACE rate in DCB-treated SV-CAD patients. The linkage of CDC42 at baseline (**A**), D1 (**B**), D3 (**C**), and D7 (**D**) with the accumulating MACE rate in DCB-treated SV-CAD patients

Additionally, no association was observed in CDC42 at baseline (*P* = 0.058) or at D1 (*P* = 0.111) with the accumulating MACE rate in DCB-treated SV-CAD patients (Fig. 4A-B). High CDC42 at D3 (*P* = 0.037) and D7 (*P* = 0.041) was linked with a lower accumulating MACE rate in DCB-treated SV-CAD patients (Fig. 4C-D).

### The ability of CDC42 to differentiate patients with MACE from those without

**Fig. 5:**
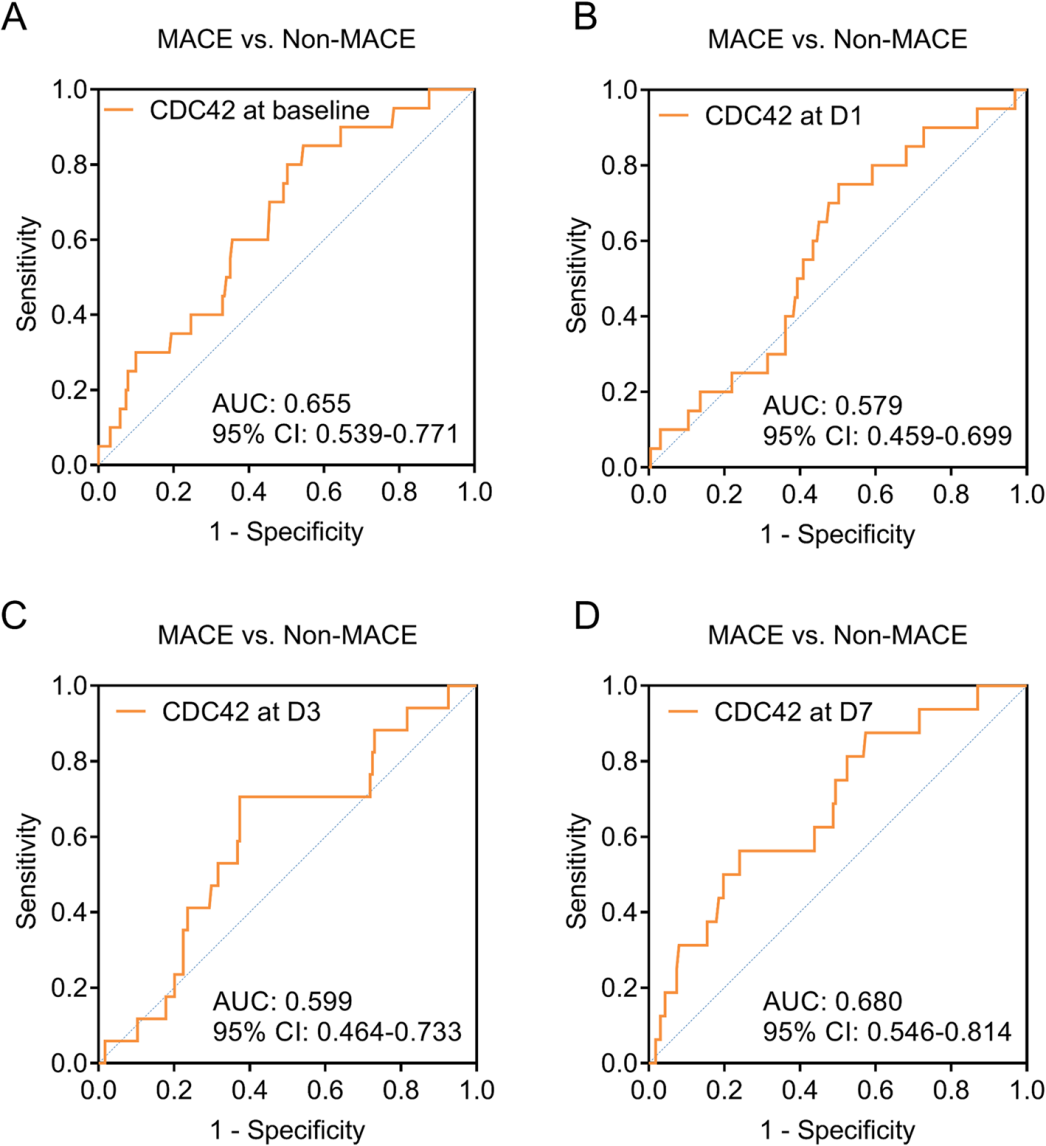
The value of CDC42 at different time points in differentiating patients with MACE from those without. ROC curves exhibiting the value of CDC42 at baseline (**A**), D1 (**B**), D3 (**C**), and D7 (**D**) to differentiate SV-CAD patients with MACE from those without

Notably, CDC42 at baseline, D1, D3, and D7 showed a certain value in discriminating patients with MACE from patients with non-MACE with the AUCs of 0.655 (95% CI: 0.539–0.771) (Fig. 5A), 0.579 (95% CI: 0.459–0.699) (Fig. 5B), 0.599 (95% CI: 0.464–0.733) (Fig. 5C), and 0.680 (95% CI: 0.546–0.814) (Fig. 5D), respectively.

### Factors related to TLF in DCB-treated SV-CAD patients

The univariate and multivariate Cox’s regression models of TLF in DCB-treated SV-CAD patients were shown in Table 4. The univariable model revealed that CDC42 at D7 (high vs. low) (hazard ratio (HR): 0.188, *P* = 0.018) was associated with a lower TLF rate, while diabetes mellitus (yes vs. no) (HR: 4.854, *P* = 0.022), multivessel disease (yes vs. no) (HR: 9.469, *P* = 0.033), and TG (high vs. low) (HR: 10.945, *P* = 0.023) were linked with higher TLF rates in DCB-treated SV-CAD patients. Next, the multivariable model found that CDC42 at D7 (high vs. low) (HR: 0.145, *P* = 0.021) and HDL-C (high vs. low) (HR: 0.081, *P* = 0.020) were independently associated with lower TLF rates, but multivessel disease (yes vs. no) (HR: 9.991, *P* = 0.032) was independently linked with a higher TLF rate in DCB-treated SV-CAD patients (Table 4).

**Table 4:** CDC42 at D7 independently predicted lower TLF in SV-CAD patients

Factors	*P* value	HR	95% CI	
			Lower	Upper
**Univariable model**				
CDC42 at baseline, high vs. low	0.068	0.236	0.050	1.113
CDC42 at D1, high vs. low	0.166	0.335	0.071	1.577
CDC42 at D3, high vs. low	0.068	0.231	0.048	1.112
CDC42 at D7, high vs. low	0.018	0.188	0.047	0.754
Age, high vs. low	0.074	4.110	0.873	19.357
Gender, male vs. female	0.244	0.471	0.133	1.672
BMI, high vs. low	0.908	1.076	0.311	3.718
History of smoke, yes vs. no	0.564	1.451	0.409	5.144
Hypertension, yes vs. no	0.217	35.651	0.122	10438.529
Hyperlipidemia, yes vs. no	0.949	0.961	0.278	3.319
Diabetes mellitus, yes vs. no	0.022	4.854	1.254	18.784
Family history of CAD, yes vs. no	0.561	0.631	0.134	2.977
Previous MI, yes vs. no	0.120	2.677	0.774	9.253
Previous PCI, yes vs. no	0.333	1.868	0.527	6.625
Previous CABG, yes vs. no	0.713	0.047	< 0.001	543892.205
Clinical manifestation, unstable angina vs. stable angina	0.645	0.743	0.210	2.633
Multivessel disease, yes vs. no	0.033	9.469	1.200	74.752
Target vessel				
LAD	Reference			
LCX vs. LAD	0.684	0.733	0.164	3.277
RCA vs. LAD	0.526	1.679	0.339	8.319
ACC/AHA type B2/C lesions, yes vs. no	0.114	60.779	0.372	9936.455
DCB diameter, high vs. low	0.197	2.771	0.588	13.050
Total length of DCB, high vs. low	0.867	0.890	0.230	3.445
WBC, high vs. low	0.874	0.905	0.262	3.128
FBG, high vs. low	0.608	1.393	0.393	4.941
Scr, high vs. low	0.483	0.635	0.179	2.255
TG, high vs. low	0.023	10.945	1.386	86.421
TC, high vs. low	0.235	2.273	0.587	8.803
LDL-C, high vs. low	0.212	2.368	0.611	9.175
HDL-C, high vs. low	0.110	0.332	0.086	1.286
CRP, high vs. low	0.089	3.840	0.815	18.092
**Multivariable model**				
CDC42 at D7, high vs. low	0.021	0.145	0.028	0.744
Multivessel disease, yes vs. no	0.032	9.991	1.216	82.093
TG, high vs. low	0.065	7.423	0.880	62.621
HDL-C, high vs. low	0.020	0.081	0.010	0.672

CDC42 was divided into high and low levels by baseline median value (500 pg/mL), and other continuous factors were divided into high and low levels by their median values

*TLF* Target lesion failure, *SV-CAD *Small-vessel coronary artery disease, *HR *Hazard ratio, *CI *Confidence interval, *CDC42 *Cell division cycle 42, *D1 *1st day of postoperation, *D3 *3rd day of postoperation, *D7* 7th day of postoperation, *BMI* Body mass index, *CAD* Coronary artery disease, *MI* Myocardial infarction, *PCI* Percutaneous transluminal coronary intervention, *CABG* Coronary artery bypass grafting, *LAD* Left anterior descending artery, *LCX* Left circumflex artery, *RCA* Right coronary artery, *ACC* American college of cardiology, *AHA* American heart association, *DCB* Drug-coated balloon, *WBC* White blood cell, *FBG* Fasting plasma glucose, *Scr* Serum creatinine, *TG* Triglyceride, *TC* Total cholesterol, *LDL-C* Low-density lipoprotein cholesterol, *HDL-C* High-density lipoprotein cholesterol, *CRP* C-reactive protein

### Factors linked with the MACE rate in DCB-treated SV-CAD patients

The univariate and multivariate Cox’s regression models of the MACE rate in DCB-treated SV-CAD patients were shown in Table 5. The univariable model showed that CDC42 at D3 (high vs. low) (HR: 0.346, *P* = 0.047) was associated with a lower MACE rate, and CDC42 at D7 (high vs. low) (HR: 0.375, *P* = 0.050) tended to be associated with a lower MACE rate in DCB-treated SV-CAD patients. Nevertheless, diabetes mellitus (yes vs. no) (HR: 2.543, *P* = 0.038), multivessel disease (yes vs. no) (HR: 4.281, *P* = 0.009), target vessel of RCA (vs. LAD) (HR: 4.494, *P* = 0.027), ACC/AHA type B2/C lesions (yes vs. no) (HR: 2.893, *P* = 0.040), TG (high vs. low) (HR: 2.855, *P* = 0.032), and CRP (high vs. low) (HR: 2.874, *P* = 0.041) were related to a higher MACE rate in DCB-treated SV-CAD patients. Afterwards, the multivariable model suggested that CDC42 at D7 (high vs. low) (HR: 0.295, *P* = 0.023) and HDL-C (high vs. low) (HR: 0.150, *P* = 0.004) were independently correlated with a lower MACE rate, but family history of CAD (yes vs. no) (HR: 3.783, *P* = 0.014), multivessel disease (yes vs. no) (HR: 4.628, *P* = 0.020), and CRP (high vs. low) (HR: 4.276, *P* = 0.029) were independently linked with a higher MACE rate in DCB-treated SV-CAD patients (Table 5).

**Table 5:** CDC42 at D7 independently predicted lower MACE in SV-CAD patients

Factors	*P* value	HR	95% CI	
			Lower	Upper
**Univariable model**				
CDC42 at baseline, high vs. low	0.066	0.408	0.157	1.063
CDC42 at D1, high vs. low	0.121	0.449	0.163	1.236
CDC42 at D3, high vs. low	0.047	0.346	0.122	0.984
CDC42 at D7, high vs. low	0.050	0.375	0.141	1.000
Age, high vs. low	0.616	1.253	0.519	3.024
Gender, male vs. female	0.933	0.957	0.348	2.636
BMI, high vs. low	0.291	1.620	0.662	3.963
History of smoke, yes vs. no	0.705	1.185	0.491	2.862
Hypertension, yes vs. no	0.079	36.046	0.662	1962.985
Hyperlipidemia, yes vs. no	0.447	1.415	0.578	3.463
Diabetes mellitus, yes vs. no	0.038	2.543	1.053	6.140
Family history of CAD, yes vs. no	0.110	2.055	0.851	4.965
Previous MI, yes vs. no	0.413	1.468	0.585	3.680
Previous PCI, yes vs. no	0.958	1.024	0.424	2.473
Previous CABG, yes vs. no	0.622	0.048	< 0.001	8655.409
Clinical manifestation, unstable angina vs. stable angina	0.868	0.925	0.369	2.319
Multivessel disease, yes vs. no	0.009	4.281	1.430	12.814
Target vessel				
LAD	Reference			
LCX vs. LAD	0.451	1.654	0.448	6.115
RCA vs. LAD	0.027	4.494	1.191	16.954
ACC/AHA type B2/C lesions, yes vs. no	0.040	2.893	1.048	7.988
DCB diameter, high vs. low	0.922	1.046	0.427	2.559
Total length of DCB, high vs. low	0.847	0.910	0.350	2.370
WBC, high vs. low	0.275	1.670	0.666	4.187
FBG, high vs. low	0.879	0.934	0.388	2.245
Scr, high vs. low	0.298	0.622	0.254	1.522
TG, high vs. low	0.032	2.855	1.097	7.433
TC, high vs. low	0.213	1.794	0.715	4.500
LDL-C, high vs. low	0.177	1.885	0.751	4.729
HDL-C, high vs. low	0.067	0.423	0.169	1.062
CRP, high vs. low	0.041	2.874	1.044	7.910
**Multivariable model**				
CDC42 at D7, high vs. low	0.023	0.295	0.102	0.848
Family history of CAD, yes vs. no	0.014	3.783	1.312	10.906
Multivessel disease, yes vs. no	0.020	4.628	1.275	16.796
HDL-C, high vs. low	0.004	0.150	0.041	0.549
CRP, high vs. low	0.029	4.276	1.159	15.772

CDC42 was divided into high and low levels by baseline median value (500 pg/mL), and other continuous factors were divided into high and low levels by their median values

*MACE* Major adverse cardiac event, *SV-CAD *Small-vessel coronary artery disease, *HR *Hazard ratio, *CI *Confidence interval, *CDC42 *Cell division cycle 42, *D1 *1st day of postoperation, *D3 *3rd day of postoperation, *D7 *7th day of postoperation, *BMI *body mass index, *CAD * Coronary artery disease, *MI *Myocardial infarction, *PCI* Percutaneous transluminal coronary intervention, *CABG* Coronary artery bypass grafting, *LAD* Left anterior descending artery, *LCX* Left circumflex artery, *RCA* Right coronary artery, *ACC* American college of cardiology, *AHA* American heart association, *DCB* Drug-coated balloon, *WBC* White blood cell, *FBG* Fasting plasma glucose, *Scr* Serum creatinine, *TG* Triglyceride, *TC* Total cholesterol, *LDL-C* Low-density lipoprotein cholesterol, *HDL-C* High-density lipoprotein cholesterol, *CRP* C-reactive protein

## Discussion

CDC42 participates in coronary artery disease by regulating atherosclerosis, systemic inflammation, and blood lipids [13, 14, 19, 20]. Specifically, CDC42 mediates endothelial regeneration and vascular recovery by the p21-activated kinase 1/protein kinase B pathway to reduce atherosclerosis [14], decreases the recruitment of macrophages and controls T helper type 17/inducible regulatory T balance to inhibit systemic inflammation [13, 19], and enhances apoA-I-mediated cholesterol efflux through adenosine triphosphate-binding cassette transporter A1 to decrease blood lipids [20]. Recent studies have disclosed the abnormal expression of CDC42 in CAD patients [10, 15]. For example, Qiang Feng et al. report that CDC42 is reduced in CAD patients compared to HCs and disease controls [10]. Mi Zhou et al. also find that CDC42 is decreased in CAD patients versus controls [15]. However, the expression of CDC42 in DCB-treated SV-CAD patients has not been explored. Similar to previous studies, our study suggested that CDC42 was descended in DCB-treated SV-CAD patients compared to HCs. Meanwhile, CDC42 exhibited a good value in discriminating DCB-treated SV-CAD patients from HCs. This might be because: (1) CDC42 alleviated atherosclerosis by regulating endothelial barrier function [21, 22]. (2) CDC42 maintained T cell homeostasis by regulating glycolysis, thereby inhibiting inflammation [13, 23]. (3) CDC42 promoted endothelial regeneration after vascular injury [11, 14]. Therefore, CDC42 reduced the risk of SV-CAD.

Moreover, the correlations of CDC42 with clinical features in CAD patients have been explored in previous studies [10, 15]. For example, Qiang Feng et al. find that CDC42 is inversely linked with the Gensini score, CRP, and the occurrence of diabetes mellitus in CAD patients [10]. Mi Zhou et al. illustrate a negative correlation of CDC42 with CRP, TC, and LDL-C in CAD patients [15]. Partly similar to the above studies, our study showed that CDC42 was negatively associated with TC, LDL-C, CRP, multivessel disease, and ACC/AHA type B2/C lesions in DCB-treated SV-CAD patients. The possible reasons were as follows: (1) CDC42 interacted with adenosine triphosphate binding cassette transporter A-I to promote cholesterol efflux in foam cells, which reduced the intracellular accumulation of cholesterol; thus, it was negatively correlated with TC and LDL-C in DCB-treated SV-CAD patients [24–26]. (2) CDC42 inhibited inflammation by adjusting the activity of macrophages and T cells, therefore it was inversely associated with CRP in DCB-treated SV-CAD patients [27, 28]. (3) CDC42 might slow the progression of SV-CAD by suppressing atherosclerosis, inflammation, and dyslipidemia; meanwhile, multivessel disease and ACC/AHA type B2/C lesions represented more complex coronary artery lesions and worse disease progression in DCB-treated SV-CAD patients [29–31]. Thus, CDC42 was negatively linked with multivessel disease and ACC/AHA type B2/C lesions in DCB-treated SV-CAD patients. In addition, our study also illustrated that CDC42 was reduced from baseline to D1 and then continuously increased from D1 to D7 in DCB-treated SV-CAD patients. This finding might be because CDC42 restrained vascular inflammation, and its expression might reflect vascular inflammation to some extent [14]. DCB-treated SV-CAD patients might have some degree of vascular trauma in the early stage of treatment, which might result in increased vascular inflammation levels, thus CDC42 was decreased from baseline to D1. With the benefits of DCB treatment, the disease conditions were gradually relieved, resulting in a reduction in systemic inflammation in patients [13]; thus, CDC42 was elevated from D1 to D7.

Although Qiang Feng et al. have shown that CDC42 can serve as a biomarker for forecasting MACE risk in CAD patients [10], the prognostic role of CDC42 in DCB-treated SV-CAD patients is not yet clear. Our study revealed that CDC42 was related to lower accumulating TLF and MACE rates in DCB-treated SV-CAD patients. The possible explanations were as follows: (1) CDC42 inhibited atherosclerosis and relieved coronary artery stenosis, thus decreasing the accumulating TLF rate [21, 22]. (2) CDC42 not only reduced the accumulating TLF rate, but also balanced blood lipid levels and reduced inflammation, thereby further reducing the accumulating MACE rate in DCB-treated SV-CAD patients [25, 27]. Furthermore, our study showed that CDC42 at D7 independently predicted TLF and MACE rates in DCB-treated SV-CAD patients. This might be because CDC42 generally increased in DCB-treated SV-CAD patients after 7 days; however, if some patients still had a low expression of CDC42 at D7, those patients might have a poor treatment efficacy of DCB, which led to a poor prognosis. Our results revealed that high CDC42 after short-term DCB treatment might better reflect the long-term TLF and MACE rates in DCB-treated SV-CAD patients.

There were several limitations in our study: (1) Our study only detected CDC42 in serum from peripheral blood samples, while previous research shows that the expression of CDC42 in peripheral blood mononuclear cells and exosomes is also important and needs to be detected in further research [32]. (2) Our study had a relatively short follow-up period (only 30 months), and future studies should evaluate the accumulating TLF and MACE rates with a longer follow-up period in DCB-treated SV-CAD patients. (3) Our study did not include disease controls. Future studies should consider enrolling disease controls to verify the expression of CDC42 in DCB-treated SV-CAD patients. (4) Our study did not use observer blinding (including blinding to patients’ clinical information or group assignments), which might cause the potential bias in the results.

In conclusion, CDC42 negatively relates to lipid levels, inflammation, multivessel disease, and ACC/AHA type B2/C lesions, whose high expression after treatment is linked with lower TLF and MACE rates in DCB-treated SV-CAD patients.

### Supplementary Information

**Additional file 1.**

## Data Availability

The datasets used and/or analysed during the current study are available from the corresponding author on reasonable request.
