# Supplementary material for: The clinical utility of circulating cell division control 42 in small-vessel coronary artery disease patients undergoing drug-coated balloon treatment
Source: BMC Cardiovasc Disord. 2023 Oct 7;23:496. doi: 10.1186/s12872-023-03476-5 (PMC10559608; doi:10.1186/s12872-023-03476-5)
Supplement: Supplementary file 1 — Additional file 1. [file 12872_2023_3476_MOESM1_ESM.docx]

**Supplementary Table 1**. Diagnostic characteristics of CDC42 between SV-CAD patients and HCs.

| Prediction  Label | SV-CAD (predicted) | Non-SV-CAD (predicted) |
| --- | --- | --- |
| SV-CAD | 174 | 37 |
| HCs | 15 | 35 |

CDC42, Cell division control 42; SV-CAD, small-vessel coronary artery disease; HCs, healthy controls.
